# Development and validation of a food and nutrition literacy questionnaire for Chinese parents of children with functional constipation (FNLQ-p)

**DOI:** 10.3389/fnut.2025.1485366

**Published:** 2025-01-31

**Authors:** Jie Yang, Hui Wang, Yanchi Shen, Hui Yang, Yan Huang, Jinjin Cao

**Affiliations:** ^1^Department of Gastroenterology, Children's Hospital of Nanjing Medical University, Nanjing, China; ^2^Department of Nursing, Nanjing BenQ Medical Center, The Affiliated BenQ Hospital of Nanjing Medical University, Nanjing, China

**Keywords:** food and nutrition literacy, questionnaire development, school-age, functional constipation, validation

## Abstract

**Objectives:**

Childhood functional constipation is a widespread condition with a global prevalence. Dietary interventions play a crucial role in the management of childhood constipation. Hence, the development and validation of a specialized food and nutrition literacy assessment tool for parents of school-aged children with functional constipation is of paramount significance.

**Methods:**

On the basis of literature review, the first draft was formed, and the results of expert correspondence and pre survey were combined to delete and modify the first draft. In the second stage, 459 parents of school-age children with constipation were invited to fill out the questionnaire. Item analysis, exploratory factor analysis, and confirmatory factor analysis were then conducted to assess the questionnaire's reliability and validity.

**Results:**

The final scale comprises 4 dimensions and 25 items. Exploratory factor analysis extracted four common factors (nutrition knowledge, nutrition skills, nutrition interaction, nutrition evaluation), and the cumulative variance contribution rate was 64. 532%. The content validity index (I-CVI) of each item level is 0.86–1, the content validity index (S-CVI) at the scale level is 0.96. The overall Cronbach'sα coefficient was 0.85. Confirmatory factor analysis supported the four-factor structure derived from exploratory analysis, with all relevant fit indices meeting standard criteria.

**Conclusions:**

The food and nutrition literacy questionnaire developed in our study had good validity and reliability, making it a useful tool for assessing the food and nutrition literacy among parents of school-aged children diagnosed with functional constipation.

## 1 Introduction

Functional constipation (FC) is one of the most common diseases in the first few years of life with a reported prevalence ranged from 1.3% to 26% (1). It is characterized by infrequent, painful, and hard stools and may be accompanied by fecal incontinence and abdominal pain (2). As a significant global public health problem, FC accounts for numerous visits to pediatric outpatients across all ages (3, 4), thus posing a considerable financial burden on families and a substantial healthcare burden on budgets. According to a 2020 report from the Bowel Interest Group, treatment of constipation cost the English National Health Service (NHS) £168 million in 2019 (5). Besides, the disease negatively impacts health-related quality of life (6, 7). On one hand, more emotional and behavioral problems were seen in children with functional constipation (8). On the other hand, parents living with FC children expressed strong feelings of isolation, and the treatment of constipation places significant psychological pressure on them (9, 10).

Diet therapy, according to the international guidelines, together with family education, toilet training, drug use and behavior change, constitutes the treatment methods for constipation (11). Recent evidence underscores the efficacy of fiber intake in improving constipation symptoms (12). Studies such as those by Tabbers (13), Loening-Baucke (14), and Quitadamo (15) have documented the beneficial effects of fiber, including glucomannan, in the dietary management of childhood constipation. Furthermore, research by Kokke (16), Weber (17), and Okuda (18) supports the improvement of FC in children and adolescents through diets enriched with fiber, vegetables, and fruits. However, research focusing on the impact of dietary factors on child constipation revealed that, compared to their non-constipated peers, children with constipation have a lower intake of fruits and vegetables (19). Moreover, two additional studies also demonstrated that, the optimal dietary composition for school-aged children, particularly in terms of fruits, vegetables, seafood, and plant proteins, remains unachieved (20, 21). Poor dietary habits such as a high-fat diet and a low-fiber diet are closely related to constipation (22). For school-aged children, who have less autonomy over their food and nutrition management than adolescents, the provision of their food and nutrition largely depends on their parents. However, a position statement regarding children's healthy diets (23) suggests that while 34% of parents aim to provide healthy food choices, they often expect children to eat whatever is available. Another 34% of parents have low expectations regarding their children's dietary habits, and 17% prioritize their child's happiness, offering only foods they are sure the child will eat, regardless of nutritional value. Some parents opt for the most convenient options without considering nutrition or the child's preferences. These approaches result in lower scores for children's healthy eating. Additionally, there's evidence that parents need support in understanding the importance of proper nutrition for maintaining health (24). Therefore, evaluating parental food and nutrition literacy (FNL) is of significant concern in managing children's functional constipation.

Food and nutrition literacy is defined as “the set of knowledge and skills which enable people to make appropriate nutrition decisions and plan, manage, select, prepare, and eat foods” (25, 26). It plays a vital role in improving the quality of an individual's diet. Food and nutritional literacy assessment is a prerequisite for assessing FNL status and proposing appropriate promotion and improvement strategies (25). Tools used in previous studies involving FNL assessment in parents of pre-school children with FC included general tools and subscales of the full scale, such as FNLQ (27), TNLAT (28), FNLQ-SC (29), CNL-E (30). However, general measuring tools are not comprehensive enough to measure the FNL of specific diseases. Up to date, there is no FNL assessment tool specifically designed for parents of school-aged children with FC.

The purpose of this study was to develop and validate the Food and Nutrition Literacy Questionnaire for Parents of School-aged Children with Functional Constipation (FNLQ-p). This initiative is aimed at providing an essential assessment tool for future efforts to evaluate and enhance the food and nutrition literacy among this population, as well as to facilitate the implementation of targeted health education programs.

## 2 Materials and methods

### 2.1 Ethical approval

Ethical approval was provided by the ethics committees of Children's Hospital of Nanjing medical University (202307006-1). We distributed the questionnaires from July 13, 2023, to January 28, 2024. All participants signed written informed consent forms.

**Figure 1:**
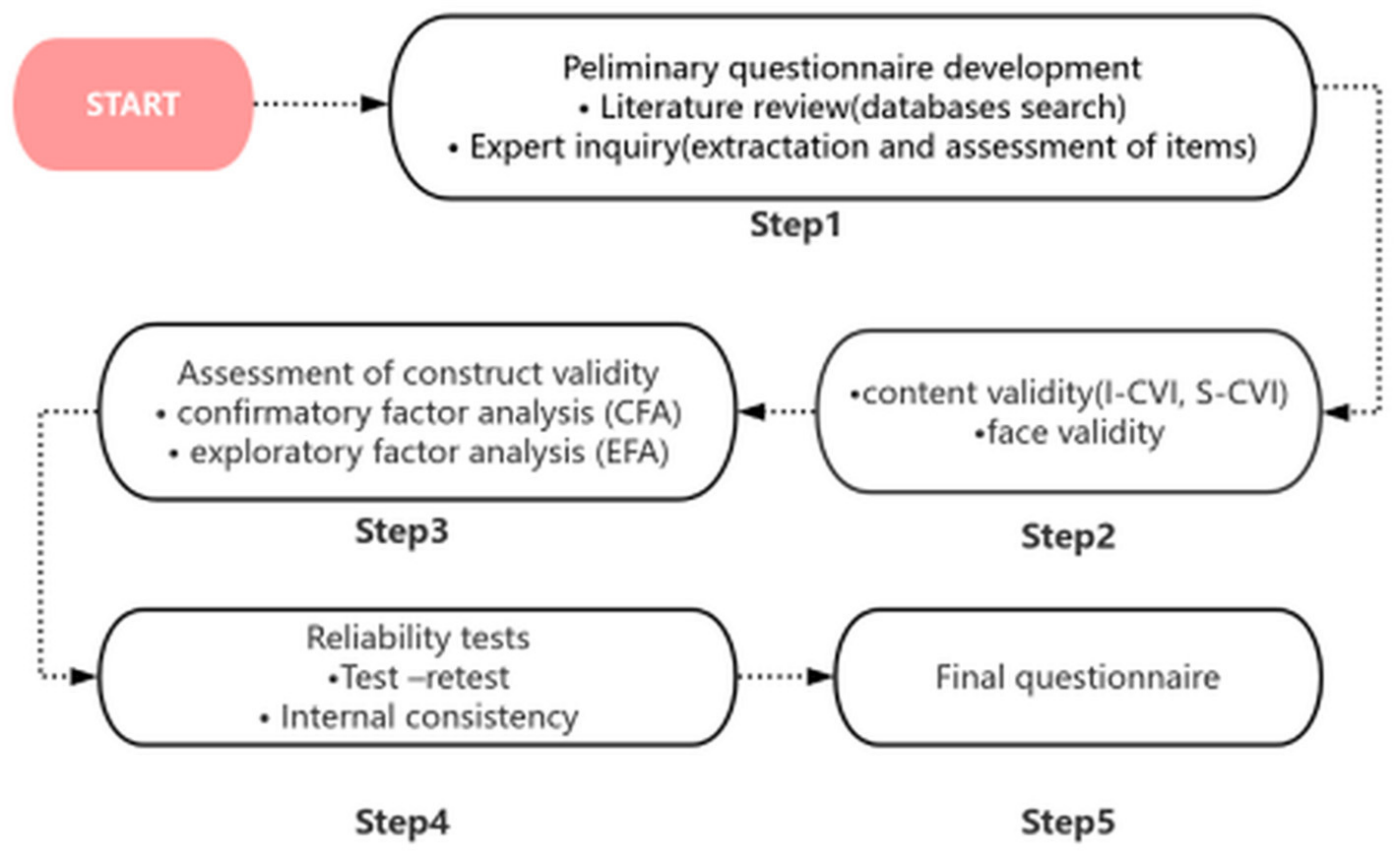
The stages of development and validation of FNLQ-p.

The procedure for the development and validation of the FNLQ-p involved multiple steps to ensure the questionnaire's scientific rigor and practicality. These steps are visually summarized in Figure 1.

### 2.2 Scale development

#### 2.2.1 Literature review

The initial scale was constructed based on a literature review guided by Nutbeam's hierarchical model of health literacy (31) and the knowledge, belief, and practice theory (32). The literature search, conducted from the inception of databases to February 2023, involved the use of databases such as Web of Science, PubMed, China National Knowledge Infrastructure (CNKI), Wanfang Database, and VIP Database. Search terms included “nutrition literacy,” “nutrition skills,” and “nutrition management.” Additional publications were identified by manually reviewing references in the included literature. Articles were selected based on their relevance to food and nutrition literacy concepts or the management of functional constipation in children. The inclusion criteria encompassed studies focusing on nutritional literacy or food-related practices in the context of managing functional constipation in children. We also included studies that examined parents' roles in shaping children's dietary habits, their nutritional knowledge, and food literacy. Only peer-reviewed articles published in English or Chinese were considered. Exclusion criteria included duplicate studies, conference abstracts, editorials, articles without accessible full text, and studies not directly related to functional constipation or relevant dietary practices. The article selection process for the literature review was conducted by three authors. JY performed the initial database search and retrieved relevant studies, HW conducted a detailed review for inclusion and exclusion criteria, and YCS cross-verified the selected articles for quality assessment and alignment with the research objectives. Discrepancies, if any, were resolved through team discussions to ensure consensus.

According to Nutbeam's hierarchical model, nutritional literacy was categorized into three parts: functional, interactive, and critical literacy (31). Functional literacy pertains to accessing, comprehending, and utilizing food and nutrition information for healthy food preparation and consumption. Interactive literacy encompasses communication, discussion, and information sharing. Critical literacy involves critical thinking and the evaluation of food and nutrition-related information. High-frequency items were identified though literature review. These items were organized, eliminating redundancies and retaining the most specific and understandable questions within the same domain. An expert group meeting in April 2023 was then convened to establish an interim questionnaire framework.

#### 2.2.2 Experts inquiry

Delphi method was used to screen the items. A total of 19 experts were selected as the inquiry subjects in April to June 2023. Two rounds of face-to-face or online mail questionnaires were issued and returned, with an interval of 2–4 weeks for each round of consultation. The inclusion criteria of experts: ①dietetics experts or nutrition education experts who have been engaged in nutrition and disease research for more than 5 years, and have an intermediate title or above; Or nursing experts who have been engaged in nursing research for more than 5 years and have the title of deputy senior or above; ②familiar with the content of nutritional literacy or the analysis of FC children's condition.

Experts were invited to evaluate the importance of each dimension and item with Likert 5-type scoring method. Each dimension and item were rated on a scale from least important to most important, with scores of 1–5, respectively. The items were screened according to the criteria of importance score ≥3.5 and coefficient of variation (CV) < 0.25. At the same time, there were columns of “modification opinion”, “addition of items and dimensions” and the overall evaluation for experts to express their opinions. Some items were supplemented and modified according to expert opinions to determine the initial scale (The general information of experts is shown in supplementary document).

#### 2.2.3 Questionnaire development

The Food and Nutrition Literacy Questionnaire for parents of FC children (FNLQ-p) was established following the steps above. The questionnaire consists of two parts: the first part collected demographic information, including age, gender, and education level of parents, as well as age, gender, type of disease, course of disease, medication, and medical history of children. The second part is the Food and Nutritional Literacy Assessment Scale for parents of FC children, comprising 25 items distributed across four dimensions: nutrition knowledge, nutrition skills, nutrition interaction, and nutrition evaluation. The scale used a 5-point Likert scale (“strongly disagree, disagree, don't know, agree, and strongly agree”). In the second part, 4 points were given for each question. The higher the score, the higher the nutrition literacy of the respondents.

### 2.3 Validation of questionnaire

#### 2.3.1 Data collection and participants

Convenient sampling was conducted in constipation specialist outpatient clinic in Children's Medical Center of Jiangsu Province from July 13, 2023 to January 28, 2024. In our study, the inclusion criteria were: (1) school-aged children between 6 to 12 years diagnosed with functional constipation according to the ROME IV criteria; (2) parents who were able to understand and complete the questionnaire. The exclusion criteria included: (1) children with significant neurodevelopmental delays or disorders; (2) children with structural gastrointestinal diseases; (3) children currently enrolled in other diet or constipation-related intervention studies.

The sample size estimation was guided by the rule of thumb: a minimum of 10 respondents per factor analysis item (33). Given the 25 items in FNLQ-p, a minimum of 250 participants was required. To ensure meaningful parameter estimates for confirmatory factor analysis, a minimum of 200 participants was needed (34). A total of 488 parents of were invited to participate, and 459 completed the entire survey, providing sufficient samples for both exploratory factor analysis (EFA) and confirmatory factor analysis (CFA) (35). Refusals were primarily due to time constraints or a lack of interest.

#### 2.3.2 Expert authority coefficient

The expert authority coefficient, denoted as Cr, reflects an expert's authority level on a specific issue or domain. This coefficient is derived from self-evaluation and is determined by two factors: the basis of judgment (Ca) and the familiarity with the issue (Cs), with the formula Cr = (Ca + Cs) / 2. An Cr ≥ 0.7 is generally considered to indicate reliable research results (36).

#### 2.3.3 Reliability tests

Reliability assessment involved internal consistency and test–retest reliability. Internal consistency was determined by calculating the Cronbach's alpha coefficient for both the overall questionnaire and individual dimensions. A coefficient >0.7 indicated acceptable reliability. To conduct test-retest reliability, 80 parents completed the questionnaire twice, with 2–4 weeks interval.

#### 2.3.4 Validity tests

An expert consultation letter was created for experts to assess each item in the initial FNLQ-p version. Seven experts, encompassing clinical nurse specialists, dietetics experts, nutrition education experts, and clinical medical experts, participated in the study. They rated item relevance and clarity on a 4-point Likert scale (1 being irrelevant/ clear to 4 being highly relevant/clear). For items rated 1 or 2, experts were asked to come up with an alternative expression. Content validity was estimated through content validity indexes at the item level (I-CVI) (reference range ≥0.78) and at the scale level (S-CVI/Ave) (reference range ≥0.90) (37). I-CVI was calculated as the number of experts giving a rating of either 3 or 4, divided by the total number of experts. S-CVI was calculated by taking an average of the I-CVIs.

We conducted cognitive interviews with 9 parents to inquire whether they understood the meaning of the items and to determine whether the content of the FNLQ-p was clear and understandable. These parents were selected through convenience sampling from the outpatient clinic of the Children's Medical Center of Jiangsu Province. Inclusion criteria included parents of school-aged children diagnosed with functional constipation who were able to understand and provide feedback on the questionnaire. These parents were not included in the study sample. During the interviews, all participants reported that they fully understood the meaning of the items and did not identify any unclear or ambiguous content. As a result, no revisions were made based on their feedback. Then a preliminary version of the 25-item and it was sent to 7 healthcare providers (including nursing assistants, nurses, and doctors) for face validity assessment.

#### 2.3.5 Statistical analysis

Statistical analysis was conducted using IBM SPSS STATISTICS 26.0 and AMOS 24.0. Descriptive statistics were employed to summarize demographic characteristics. Discrimination ability of the FNLQ-p was assessed by item-total scale correlation, and correlation coefficient below 0.3 indicating item deletions. Internal consistency reliability was measured through Cronbach's alpha coefficients, with values >0.7 considered ideal (38).

The raw data were randomly divided into two parts, part 1 (*N* = 229) for EFA to explore the factorial structure of FNLQ-p, and part 2 (*N* = 230) for CFA to confirm the results of EFA. EFA and CFA were used to examine construct validity. Prior to EFA, the sampling adequacy was tested by Kaiser-Meyer-Olkin (KMO) and Bartlett's spherical test. Factors with a factor load >0.40 and an eigenvalue >1.0 were extracted. To assess goodness of fit, the study employed chi-square/degrees of freedom (χ^2^/df, cut-off < 3), root mean square error of approximation (RMSEA, cut-off < 0.08), comparative fit index (CFI, cut-off ≥0.95), goodness-of-fit index (GFI, cut-off ≥0.85) and incremental fit index (IFI, cut-off ≥0.90).

## 3 Results

### 3.1 Scale development

In the first phase of the study, 42 projects were generated. Two rounds of expert consultation were conducted, and the recovery rates of the two rounds were 85% and 94%, respectively. The expert authority coefficients were 0.884 and 0.917, respectively. According to the expert opinion and importance score, combined with the results of group discussion, 12 items were merged, 5 items were deleted, and the content and expression of other items were modified. Finally, an initial scale with 4 dimensions and 25 items was formed.

### 3.2 Demographic characteristics

A total of 488 parents were invited to participate, and 459 completed the entire survey. The sociodemographic characteristics of the participants are shown in Table 1.

**Table 1:** Demographic characteristics of participates.

**Characteristics**	**Total (*N =* 459)**	**Reliability and validity study (*N =* 229)**
**Parents**		
**Relationship**		
Mother	439(95.6%)	218(95.1%)
Age	37.95 ± 5.45	37.49 ± 5.42
**Registered residence**		
Urban	274(59.6%)	139(60.7%)
Rural	185(40.4%)	90(39.3%)
**Working condition**		
Full-time job	282(61.4%)	141(61.5%)
Part-time job	174(37.9%)	87(37.9%)
**Children**		
Age	8.80 ± 1.94	8.79 ± 1.96
**Gender**		
Girls	239(52.1%)	123(53.7%)
**Only child**		
Yes	156(33.9%)	83(36.3%)
No	303(66.1%)	140(83.7%)
**Living at school**		
Yes	91(19.9%)	44(19.2%)
No	368(80.1%)	185(80.8%)
**Course of disease(/m)**		
≤ 12	5(1.0%)	4(1.7%)
13–17	38(8.3%)	21(9.2%)
18–23	58(12.6%)	29(12.9%)
≥24	356(77.6%)	173(75.5%)

The sum of percentages did not add up to 100.00% because of the default value.

### 3.3 Reliability

The total Cronbach's α coefficient of the scale was 0.850. The Cronbach's α coeficients for the 4 domains of the FNLQ-p were 0.831, 0.906, 0.892, and 0.906, respectively.

### 3.4 Content and face validity

Analyses showed that I-CVI values of the FNLQ-p ranged from 0.86 to 1, and the S-CVI was 0.96, indicating acceptable content validity. In addition, experts recognized the clarity of the content. They generally believed that FNLQ-p was easy to understand and had an appropriate entry length.

### 3.5 Construct validity

The total correlation coefficients of 25 items ranged from 0.362 to 0.593, which were >0.3, within the acceptable range and were statistically significant. Deleting any item will result in a decrease in the Kronbach coefficient, indicating that 25 items are suitable for exploratory factor analysis.

After project analysis, exploratory factor analysis was conducted on 25 items. The Kaiser-Meyer-Olkin test of sample validity returned a value of 0.797, and Bartlett's test of sphericity showed a significant difference (χ^2^= 3,951.84, *p* < 0.001), indicating that factor analysis was applicable. Using 0.4 as the critical criterion for factor loading, EFA suggested a four-factor solution, which explained 64.53% of the total variance. The factor loads of the four-factor model of the FNLQ-p ranged between 0.677 and 0.885. Table 2 details which items loaded on the four-factor.

**Table 2:** Item-total correlation, reliability coefficients and factor loads of the FNLQ-p (*N* = 229).

**Factor name**	**Item**	**Item-total correlation**	**Factor loads**	**Cronbach's alpha if item deleted**	**Cronbach's alpha**
Knowledge					0.831
	I know that proper diet and nutrition are important measures for constipation treatment	0.362^**^	0.768	0.847	
	I am concerned about the amount of fiber in my child's diet	0.488^**^	0.844	0.843	
	I am concerned about the nutrient content of food	0.381^**^	0.726	0.847	
	I understand the classification, sources and main nutritional properties of food	0.412^**^	0.795	0.846	
	I know the proper amount of fiber my child needs every day	0.444^**^	0.730	0.845	
Skill					0.906
	I would put my child on a diet that helps relieve symptoms, whether he likes it or not	0.503^**^	0.798	0.843	
	I would encourage and supervise my children eat more fruits and vegetables	0.554^**^	0.745	0.841	
	I would provide children with fruit or juice rich in sorbitol	0.559^**^	0.804	0.841	
	I would keep a nutrition diary for my children	0.476^**^	0.728	0.844	
	I can store and cook food in a proper way	0.580^**^	0.823	0.840	
	I can match the food reasonably	0.579^**^	0.777	0.840	
	I can understand the nutritional information on the food lable	0.593^**^	0.774	0.839	
	I can judge the quality of food and choose fresh and healthy food	0.494^**^	0.763	0.843	
Interaction					0.892
	I can quickly retrieve the nutrition and diet information related to children's diseases through the Internet	0.417^**^	0.782	0.846	
	I can accept reasonable food and nutrition suggestions from my family or friends	0.402^**^	0.835	0.846	
	I can help children with the same disease to formulate a reasonable diet plan	0.465^**^	0.883	0.844	
	I can discuss the child's diet with professionals (community doctors, nurses, etc.)	0.392^**^	0.677	0.847	
	I can actively spread the knowledge and skills of food and nutrition related to diseases to others	0.461^**^	0.770	0.844	
	I can easily refuse food recommended by my family or friends that is not conducive to children's disease	0.467^**^	0.869	0.844	
Evaluation					0.906
	I can judge whether daily diet is conducive to disease	0.388^**^	0.806	0.848	
	I can judge whether the nutrition information disseminated by various media (internet, newspapers, magazines, etc.) is scientific and reasonable	0.482^**^	0.756	0.844	
	I can make a correct judgment based on its nutritional value and children's diseases in the face of all kinds of food	0.412^**^	0.849	0.846	
	I can express my opinions and opinions clearly when communicating with professionals	0.460^**^	0.858	0.845	
	I can consider the suitability of the food nutrition advice provided by the medical staff	0.430^**^	0.823	0.846	
	I can make the right judgments based on my child's condition in the face of fortified foods or supplements	0.400^**^	0.885	0.846	
Total					0.850

^**^p, level of statistical of significance; significant (*p* < 0.01).

**Figure 2:**
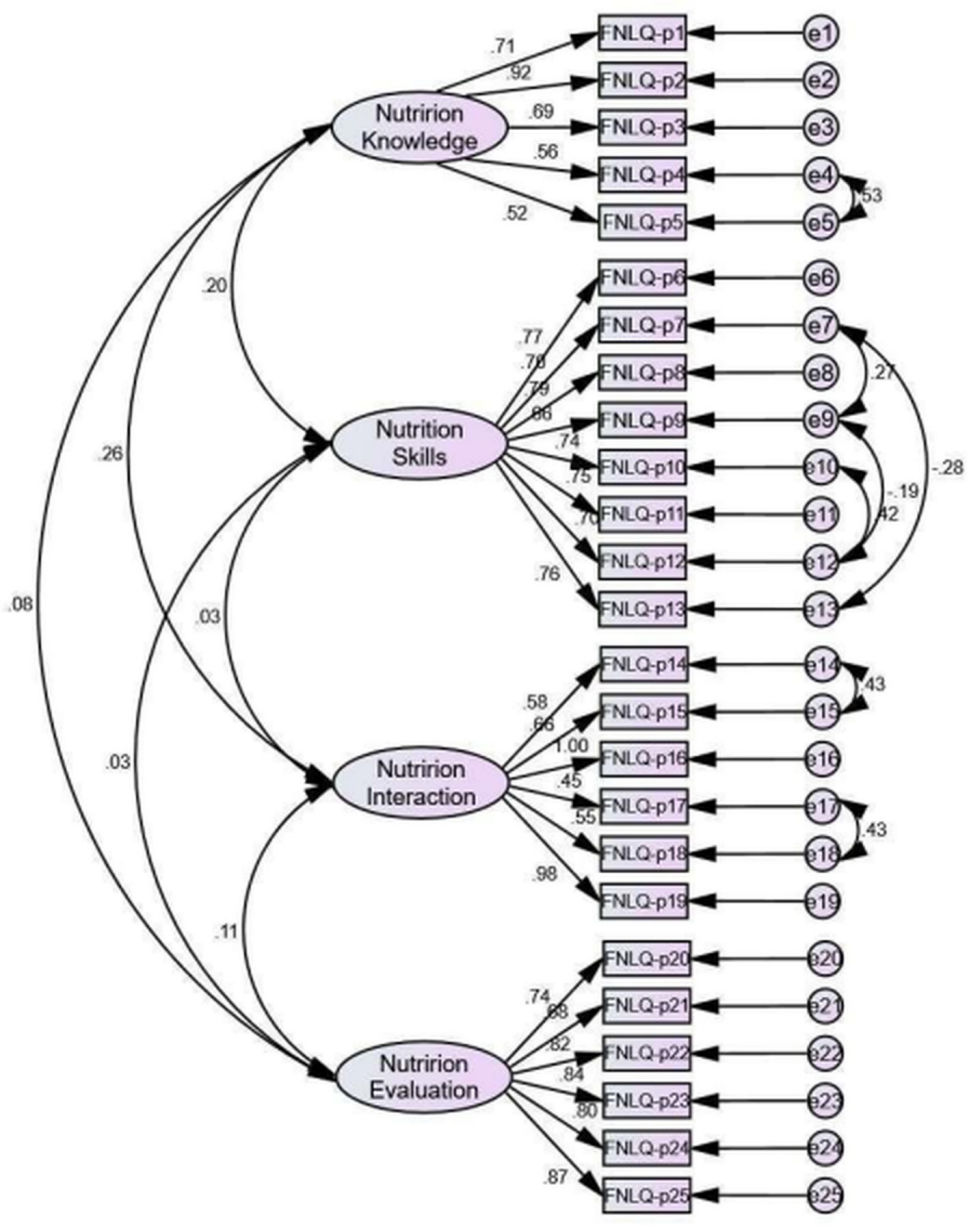
CFA of the modified four-factor model of the FNLQ-p (*N* = 230).

CFA was performed to examine the four-factor model with part 2 data (*N* = 230). The primary fitting index weren't meet the fitting standard. Based on goodness-of-fit statistics, the adjusted four-factor model were acceptable (Figure 2). Table 3 shows the model fit indices of the FNLQ-p for the primary and secondary models.

**Table 3:** Fit indices of the models (*N* = 230).

**Fit indices**	**Good fit**	**Acceptable fit**	**Four-factor model**	**Adjusted four-factor model**
χ^2^/*df*	1 < χ^2^/*df* < 3	3 < χ^2^/*df* < 5	3.095	2.274
RMSEA	0 ≤ RMSEA ≤ 0.05	0.05 < RMSEA < 0.08	0.096	0.075
CFI	0.97 ≤ CFI ≤ 1	CFI ≥ 0.90	0.853	0.913
GFI	0.90 ≤ GFI ≤ 1	0.85 ≤ GFI < 0.90	0.781	0.841
IFI	0.95 ≤ IFI ≤ 1	IFI ≥ 0.90	0.854	0.914
TLI	0.95 ≤ TLI ≤ 1	TLI ≥ 0.90	0.836	0.900

χ^2^/df, chi-square/degree of freedom; RMSEA, root mean square error of approximation; CFI, comparative fit index; GFI, goodness of fit index; IFI, incremental fit index, TLI, tucker-lewis index.

## 4 Discussion

This questionnaire was developed on the basis of Nutbeam's hierarchical model, with reference to relevant literature and guidelines. The scale consists of four aspects: nutrition knowledge, nutrition skills, nutrition interaction and nutrition evaluation. The authority coefficients of experts were 0.884 and 0.917, respectively, indicating that the authority of experts was high and the results of correspondence consultation were reliable. The analysis results of items showed that the correlation coefficients between the total score of items and scale were all >0.4, and the Cronbach's a coefficient did not increase after deleting all items, indicating good differentiation and homogeneity between items and dimensions. Content validity index and factor analysis were used to evaluate the scale validity. S-CVI and I-CVI were 0.96 and 0.86–1, indicating good content validity. A total of 4 factors were extracted from exploratory factor analysis, accounting for 66.532% of variance. The number of factors extracted was basically consistent with the preset dimensions. Cronbach's α coefficient was used to describe the internal consistency reliability of the scale. Cronbach's α coefficient of the total scale was greater than 0.80, and Cronbach's α coefficient of the subscale was >0.7, indicating good reliability. In this study, the Cronbach's α coefficient of the total scale was 0.850, and the Cronbach's α coefficient of each dimension was 0.831–0.906, indicating that the scale had good internal consistency. To sum up, the scale is scientific. The development of the scale follows a strict psychometric procedure.

Many current nutritional literacy assessment tools do not cater specifically to children with constipation. Liu et al. developed a Food and Nutrition Literacy Questionnaire for Chinese school-aged children, validating it among children aged 7–19 years (29). Their questionnaire focuses more on children's perspectives on dietary behaviors and recollection of previously consumed foods. It must be acknowledged that parents, as food providers and regulators, play a crucial role, especially since children have relatively less autonomy in their diet compared to adolescents. A study from Nigeria showed that a mother's nutritional knowledge is closely linked to her child's nutritional outcomes, and enhancing maternal nutritional literacy could improve children's nutrition and reduce malnutrition (39). Iranian researchers faced similar challenges with a scale developed to assess food and nutrition literacy among elementary students (40). Gibbs et al. highlighted the decisive role of parents in the development of children's eating behaviors, revising the Nutrition Literacy Assessment Instrument and validating its reliability and validity, aiming to provide new insights into addressing childhood obesity (41). This underscores the importance of developing disease-specific scales. Given the significant role of food and nutrition in gastrointestinal diseases (42), the knowledge of nutritional intake, rationality in dietary structure, dietary compliance, and information interaction capabilities of parents of children with functional constipation are crucial in the management and prevention of their children's condition. The evaluation of parents' food nutrition literacy of children with functional constipation should be disease and age specific. In terms of diseases, “I am concerned about the amount of fiber in my child's diet”, “I would provide children with fruit or juice rich in sorbitol”, “I would encourage and supervise children to eat more fruits and vegetables” and other items in this study involve specific dietary needs of diseases, which has good specificity for the assessment of food and nutritional literacy of parents of children with constipation. In terms of age, parents are the managers of children's diet, the scale constructed in this study includes items such as “I would put my child on a diet that helps relieve symptoms, whether he likes it or not” and “I would keep a nutrition diary for my children”, which are child specific. A survey from Australia on nutritional information demands among parents of elementary students revealed that 51% of parents wished to acquire more knowledge about food and nutrition (43). Our developed questionnaire comprehensively assesses the food and nutrition literacy of parents of children with functional constipation across four dimensions: knowledge, skills, communication, and evaluation. By providing a scientifically validated tool, based on Nutbeam's hierarchical model of health literacy, we enable clinicians and nutrition educators to assess and enhance the food and nutrition literacy among this specific parent population. This, in turn, empowers parents with the knowledge and skills needed to make informed dietary decisions that can alleviate the symptoms of functional constipation in their children.

The specificity and reliability of the FNLQ-p, as evidenced by its strong psychometric properties, underscore its potential to significantly impact the clinical approach to pediatric constipation, promoting a more nuanced understanding of dietary management strategies. Ultimately, the implementation of this questionnaire in clinical and educational settings is expected to contribute to improved health outcomes and quality of life for children suffering from functional constipation.

We acknowledge that while our initial validation process was rigorous, including expert feedback and statistical analysis, a limitation of our study is the absence of comparative validation with existing validated questionnaires. Future research will seek to address this by exploring comparative validation studies, which could offer additional validation and context for the FNLQ-p's effectiveness. This approach will further our tool's refinement and support its application in clinical settings. Secondly, our study utilized convenience sampling, which, while effective for initial exploration, introduces potential biases that may affect the generalizability of our findings. The disproportionate representation of mothers over fathers in our sample is noteworthy, as it may influence the reported levels of nutrition literacy and its impact on dietary management practices for children with FC. Future studies could benefit from employing stratified sampling methods to ensure a more balanced representation of both mothers and fathers, thereby providing a more nuanced understanding of parental roles in managing functional constipation. Lastly, our study did not delve into the various factors that might influence the food and nutrition literacy among Chinese parents of children diagnosed with FC. Understanding these influencing factors is critical for tailoring more effective nutritional education and intervention programs. In future research, a comprehensive analysis of demographic, socioeconomic, and cultural factors that may affect parental nutrition literacy and their capacity to manage their child's condition could provide invaluable insights. This exploration will enhance the applicability of the FNLQ-p by informing the development of targeted educational materials and interventions.

## Data Availability

The raw data supporting the conclusions of this article will be made available by the authors, without undue reservation.
